# Improved Adherence to Antiretroviral Therapy Observed Among HIV-Infected Children Whose Caregivers had Positive Beliefs in Medicine in Sub-Saharan Africa

**DOI:** 10.1007/s10461-016-1582-8

**Published:** 2016-10-19

**Authors:** G. Abongomera, A. Cook, V. Musiime, C. Chabala, M. Lamorde, J. Abach, M. Thomason, V. Mulenga, A. Kekitiinwa, R. Colebunders, C. Kityo, A. S. Walker, D. M. Gibb

**Affiliations:** 1 Joint Clinical Research Centre, Gulu, Uganda; 2 Faculty of Medicine, University of Antwerp, Antwerp, Belgium; 3 Medical Research Council Clinical Trials Unit at University College London, London, UK; 4 Joint Clinical Research Centre, Kampala, Uganda; 5 Faculty of Paediatrics, Makerere University College of Health Sciences, Kampala, Uganda; 6 University Teaching Hospital, Lusaka, Zambia; 7 Infectious Diseases Institute, Kampala, Uganda; 8 Baylor-Uganda, Kampala, Uganda

**Keywords:** Adherence, Children, Beliefs in medicine, Sub-Saharan Africa, Antiretroviral therapy

## Abstract

A high level of adherence to antiretroviral treatment is essential for optimal clinical outcomes in HIV infection, but measuring adherence is difficult. We investigated whether responses to a questionnaire eliciting caregiver beliefs in medicines were associated with adherence of their child (median age 2.8 years), and whether this in turn was associated with viral suppression. We used the validated beliefs in medicine questionnaire (BMQ) to measure caregiver beliefs, and medication event monitoring system caps to measure adherence. We found significant associations between BMQ scores and adherence, and between adherence and viral suppression. Among children initiating Antiretroviral therapy (ART), we also found significant associations between BMQ ‘necessity’ scores, and BMQ ‘necessity-concerns’ scores, and later viral suppression. This suggests that the BMQ may be a valuable tool when used alongside other adherence measures, and that it remains important to keep caregivers well informed about the long-term necessity of their child’s ART.

**Electronic supplementary material:**

The online version of this article (doi:10.1007/s10461-016-1582-8) contains supplementary material, which is available to authorized users.

## Introduction

The treatment of HIV with antiretroviral drugs requires good adherence, which can be challenging for children and caregivers in low-income countries [1, 2]. Known barriers include transport to clinics and long waiting times once there, while food insecurity, caregiver illness and elderly non-parent caregivers can pose additional problems [3–6]. Suggested ways to improve adherence include the use of peer counsellors, patient-selected treatment supporters, family focused treatment, and financial support for food and transport [7–10]. However, the feasibility and sustainability of these measures is often limited.

Despite the problems, high levels of antiretroviral therapy (ART) adherence have been reported among patients in sub-Saharan Africa, sometimes higher than those in high-income countries such as the United States of America [11, 12]. Self-reported adherence levels among patients purchasing ART in Kampala were comparable with those found in a high-income country [13]. The challenge is therefore to identify patient groups most likely to have poor adherence, and, particularly to identify caregivers whose children may be at risk of poor adherence given that HIV-infected children will need to take ART for life.

The monitoring of adherence by clinics is difficult. The CHAPAS-1 study compared caregiver report, pill counts, Medication Event Monitoring System (MEMS) caps and a visual analogue scale, finding that only adherence as measured by MEMS caps was significantly associated with viral suppression [14]. Unfortunately, while MEMS caps may provide the most precise measure of adherence, they are also expensive and sometimes considered intrusive [15, 16].

The ‘necessity-concerns’ framework has been proposed as a model for understanding patients’ perspectives on ART, and hence for predicting treatment uptake and adherence to treatment once started [17, 18]. Experience in a variety of disease areas has shown that factors affecting patients’ medication decisions fall into two categories: perceptions of ‘necessity’ for treatment, and ‘concerns’ of potential adverse effects that might include long term toxicity or lifestyle disruption [19]. The Beliefs about Medicines Questionnaire (BMQ) is a validated tool that separately measures ‘necessity’ and ‘concern’ [20]. Patients with strong beliefs in ‘necessity’ are likely to have higher treatment adherence, while those with strong ‘concerns’ are at risk of lower adherence. By combining the two measures in a ‘necessity-concerns’ score, a single score is produced that balances the opposing tensions within each patient. We used the BMQ to measure the children’s caregivers beliefs about medication prescribed for their children in this study.

In this study we investigated two hypotheses: (1) that caregiver beliefs in medicine were associated with their child’s adherence; (2) that adherence was associated with subsequent virological outcomes. If supportive evidence was found for both these hypotheses it may suggest that the BMQ is a feasible method for clinics to identify children at risk of treatment failure because of poor adherence, and enable support to be targeted appropriately and efficiently.

## Methods

This study was part of the CHAPAS-3 randomised controlled clinical trial; it was a sub-study planned at the start of the main trial (ISRCTN Trial Registry number, 69078957).

### Main Study

The CHAPAS-3 trial was an open-label, parallel-group randomised trial comparing three nucleoside reverse transcriptase inhibitors (NRTIs) in first-line treatment of HIV-infected African children in three centres in Uganda and one in Zambia (2010–2014). 478 children were randomised to receive stavudine (d4T), zidovudine (ZDV) or abacavir (ABC), together with lamivudine (3TC) and either efavirenz (EFV) or nevirapine (NVP). Drugs were provided as dual-NRTI plus single non-NRTI (NNRTI), or as triple drug scored tablet ‘mini-pill’ formulations. Children were ART naïve or ART experienced (on ART for more than 2 years with viral load <50 copies/ml); follow-up was for at least 96 weeks. The primary endpoint was grade 2/3/4 clinical or grade 3/4 laboratory adverse events with efficacy as measured by viral load suppression being a secondary outcome (Grading of adverse events: grade (1) mild, no intervention; grade (2) moderate, minimal intervention; grade (3) severe, hospitalisation indicated; grade (4) potentially life-threatening, urgent intervention indicated). The trial results showed no difference in toxicity or efficacy between the three NRTIs, but endorsed the WHO 2013 guidelines recommendation of once daily ABC-containing regimen for African children due to absence of hypersensitivity and superior resistance profile [21].

### Adherence Substudy

The substudy aimed to investigate whether caregiver’s views on medicines prescribed for their children affected adherence to ART given to the child, and ultimately their treatment outcome. The BMQ was used to measure caregivers attitudes towards their child’s treatment, adherence was measured using MEMs caps and HIV-1 viral load assayed with Roche COBAS Ampliprep/Taqman v2.0.

The BMQ is an 18-item validated instrument, with ten *specific* questions measuring beliefs about the actual medicines being prescribed to the patient (here modified to relate to the caregiver’s child), and eight questions of wider scope that measure *general* beliefs about all medicine [17–20]. Five of the *specific* questions are used to calculate the ‘necessity’ score and the other five used to calculate the ‘concern’ score. The ‘necessity-concern’ score therefore derives from the *specific* questions and directly relates to medicine currently prescribed. Four of the *general* questions are used to calculate a score measuring beliefs in the ‘overuse’ of medicine and the other four to calculate a score on ‘perceived harm’ done by medicine. Two further questions were added, of interest in CHAPAS-3, eliciting information on treatment side effects and on whether divine healing was considered more important than medicine. Scoring of the questionnaires is described in supplementary material 1.

MEMS caps record the date/time of every pill bottle opening using an electronic chip contained in the cap. In CHAPAS-3, children used MEMS caps during weeks 0–18 and weeks 54–72, with caps rotating between different children as the study progressed. Adherence data was thus collected for a child’s first 4 months in the study (start of treatment for ART naïve), and for another 4 months starting at 1 year. The calculation of adherence from MEMS data is described in supplementary material 2.

### Statistical Analysis

ART naïve and ART experienced children were analysed separately since ART experienced children were expected to have good adherence, all having been on treatment for at least 2 years and with suppressed viral load. Analysis was also conducted separately for the first four-month period (Period 1), and the 4 months starting at 1 year (Period 2), this was to investigate behaviour at the time children initiated treatment, and then to see if any changes had occurred after 1 year’s experience of treatment. BMQ and MEMS data were used to examine our first hypothesis that caregiver beliefs in medicine were associated with their child’s adherence, while MEMS data and viral load were used to examine our second hypothesis that adherence was associated with subsequent virological outcomes. Analysis of Period 1 included children with MEMS data in weeks 0–18, BMQ data at week 0, 6 or 24, and viral load measured at week 48. Analysis of Period 2 included children with MEMS data in weeks 54–72, BMQ data at week 48 or 72, and viral load measured at week 96. The lower limit of detection for viral load assays was 100 copies/ml because these assays were run retrospectively and many samples had low volumes and therefore had to be diluted.

Univariate associations were investigated between BMQ scores, MEMS adherence and viral load with baseline (trial enrolment) covariates including age, sex, relation to primary carer, treatment arm, CD4 % and weight-for-age Z-score (WAZ).

Multivariate repeated-measures regression models were used to explore the association between BMQ and MEMS, between MEMS and VL, and between BMQ and VL. Linear regression models were used for continuous outcomes of BMQ and MEMS, logistic models for the binary outcome of VL suppression. These models were adjusted for baseline covariates where a significant univariate association (p < 0.05) had been identified with either BMQ, MEMS or VL.

## Results

In Period 1, 271 (74 %) ART naïve and 97 (86 %) ART experienced children with BMQ, MEMS and viral load data were included in the analysis (Table 1). Among ART naïve children, median age was 2.8 years, 53 % were male, median CD4 % was 20 and 49 % had WHO stage 3/4 disease. ART experienced children were older with signs of CD4 recovery after median 3.5 years treatment; their median age was 6.2 years and median CD4 % was 35 %. Demographic and disease characteristics were similar to those of the overall CHAPAS-3 population [21]. Analysis of Period 2 included 235 ART-naïve and 98 ART-experienced children, 70 and 88 % of those in follow-up respectively. Missing data were primarily due to MEMS cap failures, meaning there were insufficient caps available for all children in the study in each time period. MEMS cap failures appeared to occur at random.

**Table 1:** Follow-up and demographic characteristics

	Naive at enrolment	Experienced at enrolment
Period 1	(n = 365)	(n = 113)
Children in follow-up	365 (100%)	113 (100%)
With MEMS, BMQ and VL	271 (74)%	97 (86)%
Baseline characteristics	n = 271	n = 97
Age, years	2.8 (1.6,3.9)	6.2 (5.5–7.2)
Sex, male	144 (53%)	49 (51%)
WHO stage		
1	32 (12%)	24 (25%)
2	105 (39%)	20 (21%)
3	115 (42%)	36 (37%)
4	19 (7%)	17 (18%)
CD4%	20% (13–25%)	35% (30–39%)
Weight-for-age Z	−1.9 (−3.0 to −0.9)	−1.1 (−1.7 to −0.3)
Primary carer, mother	201 (74%)	59 (61)%
Treatment arm		
D4T	99 (37%)	28 (29%)
ZDV	79 (29%)	39 (40%)
ABC	93 (34%)	30 (31%)
Period 2		
Children in follow-up	335 (100%)	112 (100%)
With MEMS, BMQ and VL	235 (70%)	98 (88%)

Numbers show n (%) or median (IQR)

### Descriptive Summary of BMQ, MEMS and Viral Load Data

Responses to the BMQ indicated a positive overall attitude among caregivers towards treatment (Table 2). Strong beliefs in the necessity of treatment were expressed with a median score of 20 (IQR 19.3,21.7, possible range 5–25). Beliefs in necessity considerably outweighed concerns about treatment, giving highly positive ‘necessity-concern’ differential scores (ART naïve Period 1, median = 8.3, IQR 6.7–9.7). For the ‘general’ belief scales, overuse and harm, carers on average had neutral views about overuse, but expressed some disagreement with statements that medicines caused harm. For these scales the mid-point was 12, median overuse score was 12.3 among ART naïve in Period 1 (IQR 11, 13.7). Some positive change in scores was also observed between Periods 1 and 2, particularly among those ART naïve at enrolment, belief in necessity increased while concern reduced, increasing further the necessity-concern differential (median = 8.3 vs. 10.0 in Periods 1 and 2 respectively, p < 0.001).

**Table 2:** Descriptive statistics

	Naive at enrolment		Experienced at enrolment		
Period 1	(n = 271)		(n = 97)		
BMQ					
Necessity^a^	20.0 (19.3–21.7)		21.0 (20.0–22.3)		
Concern^a^	12.0 (10.7–14.7)		11.7 (10.3–13.7)		
Necessity-concern^b^	8.3 (6.7–9.7)		9.3 (7.3–10.7)		
Overuse^c^	12.3 (11.0–13.7)		11.3 (10.3–13.0)		
Harm^c^	10.0 (9.0–11.0)		9.3 (8.3–10.7)		
MEMS					
Days with data	127 (125–127)		127 (127–127)		
Adherence	92.0% (83.9–96.0%)		94.9% (87.4–98.4%)		
VL					
<100 copies/ml	186 (69%)		90 (93%)		
Period 2	(n = 235)	P^d^	(n = 98)		P^d^
BMQ					
Necessity^a^	20.5 (20.0–21.5)	<0.001	20.5 (20.0–22.5)		0.78
Concern^a^	10.5 (10.0–12.0)	<0.001	11.0 (10.0–13.0)		0.002
Necessity-concern^b^	10.0 (8.0–11.0)	<0.001	10.0 (6.5–11.5)		0.16
Overuse^c^	11.0 (10.0–12.5)	<0.001	10.3 (9.5–12.0)		0.002
Harm^c^	9.5 (8.5–11.0)	0.01	9.0 (8.0–10.5)		0.78
MEMS					
Days with data	127 (124–127)		127 (125–127)		
Adherence	96.5% (85.8–98.4%)	0.006	92.5% (80.3–98.0%)		<0.001
VL					
<100 copies/ml	174 (74%)		94 (96%)		

Numbers show median (IQR) or n(%)

*BMQ* beliefs in medicine questionnaire, *MEMS* medication event monitoring system adherence, Viral load <100 copies/ml

^a^Range 5–25, midpoint 15

^b^Range -20–20, midpoint 0

^c^Range 4–20, midpoint 12

^d^Wilcoxon paired signed rank test comparing periods 1 and 2 in children with measurements at both timepoints (216 naïve, 86 experienced)

MEMS data indicated a high level of adherence to treatment, on average over 90 % of doses were taken among ART naïve and experienced groups in Periods 1 and 2 (Table 2). Observed viral load suppression was consistent with good adherence to treatment, 69 % of children ART naïve at enrolment were suppressed <100 copies/ml by week 48, 93 % of ART experienced children remained suppressed. Corresponding figures in Period 2 (VL at week 96) were 74 and 96 % of ART naive and experienced children respectively. Some positive change was again seen between Periods 1 and 2 in those ART naïve at enrolment, with higher adherence in Period 2 (92 vs. 96.5 % in Periods 1 and 2 respectively, p = 0.006), although a small decline in adherence was observed in the ART experienced group.

Statistically significant (p < 0.05) associations were observed between several baseline demographic and disease-related covariates, and either BMQ necessity-concern scores, MEMS adherence or viral suppression in Period 1 (Table 3). Among ART-naïve children, increased viral suppression was observed in girls, older children and those with higher WAZ. BMQ scores were associated with both WHO stage and CD4 %, while MEMS adherence was better in children with high WAZ. Both BMQ scores and MEMS adherence also differed between centres. Since all covariates showed some association with BMQ scores, MEMS adherence or viral suppression, multivariate models were adjusted for all the factors considered.

**Table 3:** Univariate associations of baseline characteristics with BMQ and MEMS (linear regression), and viral suppression <100 copies/ml (logistic regression) BMQ at week 0, 6 or 24, MEMS adherence to week 18, Viral load at week 48

	BMQ (necessity−concern)			MEMS			VL		
	β	95% CI	p	β	95% CI	p	β	95% CI	p
Naive at enrolment									
Age (yrs)	−0.09	(−0.26–0.08)	0.28	0.45	(−0.12–1.01)	0.12	**1.22**	**(1.04–1.44)**	**0.01**
Sex (female)	0.21	(−0.48 to 0.89)	0.55	0.71	(−1.57–2.99)	0.54	**1.86**	**(1.10–3.15)**	**0.02**
Main carer (mother)	−0.65	(−1.42v0.12)	0.10	1.04	(−1.55–3.63)	0.43	1.20	(0.67–2.13)	0.54
WHO stage ¾	**0.74**	**(0.06–1.41)**	**0.03**	−0.79	(−3.06–1.48)	0.49	1.32	(0.79–2.21)	0.29
CD4%	**0.07**	**(0.04–0.10)**	**<0.01**	−0.10	(−0.21–0.01)	0.07	1.01	(0.99–1.04)	0.39
WAZ	−0.19	(−0.42 to 0.03)	0.09	**0.80**	**(0.06–1.55)**	**0.03**	**1.24**	**(1.05–1.47)**	**0.01**
Centre^a^						**<0.01**			0.71
Lubowa	0.13	(−0.75 to 1.02)		5.74	(2.74–8.74)		0.73	(0.36–1.49)	
Baylor	−1.50	(−2.43 to −0.57)		3.94	(0.79–7.09)		0.86	(0.40–1.84)	
Gulu	1.06	(−0.01 to 2.12)		0.11	(−3.48–3.71)		0.64	(0.27–1.47)	
Experienced at enrolment									
Age (yrs)	0.23	(−0.09 to 0.54)	0.16	−0.78	(−1.81–0.24)	0.13	0.92	(0.62–1.38)	0.70
Sex (female)	−0.12	(−1.26 to 1.03)	0.84	1.21	(−2.50–4.91)	0.52	2.61	(0.48–14.2)	0.27
Main carer (mother)	**−1.61**	**(−2.73 to−0.48)**	**0.01**	−0.32	(−4.13–3.49)	0.87	1.18	(0.25–5.58)	0.84
WHO stage ¾	0.35	(−0.79 to 1.50)	0.54	−1.43	(−5.15–2.29)	0.45	0.18	(0.02–1.57)	0.12
CD4%	0.01	(−0.06 to 0.09)	0.73	0.22	(−0.02–0.46)	0.07	0.99	(0.89–1.09)	0.78
WAZ	−0.28	(−0.84 to 0.29)	0.33	−1.20	(−3.02–0.63)	0.20	0.66	(0.28–1.53)	0.33
Centre^a^			**0.05**			0.33	^b^		
Lubowa	0.16	(−1.28 to 1.60)		3.54	(−1.23–8.31)				
Baylor	−1.42	(−2.70 to −0.14)		0.78	(−3.47–5.02)				

Associations statistically significant at the 5 % level shown in bold

^a^Reference centre is UTH, Zambia

^b^Model not fitted due to empty cells

### BMQ and MEMS Adherence (Hypothesis 1)

High BMQ necessity-concern scores were significantly associated with better adherence in ART-naïve children (Table 4); in Period 1a difference of +1 point in necessity-concern was associated with a 0.33 % increase in adherence (p = 0.002). No significant association was observed between BMQ harm and overuse scores and adherence. Some evidence of poorer adherence was seen among ART-naïve children whose carers reported side effects of treatment (p = 0.09), while significantly worse adherence was observed in Period 2 among ART naïve children whose carers believed in divine healing (p = 0.008).

**Table 4:** Association between MEMS data andBMQ scores, adjusted for baseline characteristics

	Naive at enrolment			Experienced at enrolment		
	Diff	95 % CI	p	Diff	95 % CI	p
Period 1	(n = 271)			(n = 97)		
BMQ						
Necessity-concern	**0.33**	**(0.12–0.54)**	**0.002**	0.04	(−0.31–0.39)	0.83
Necessity	**0.45**	**(0.05–0.84)**	**0.03**	**0.64**	**(0.04–1.23)**	**0.04**
Concern	**−0.30**	**(−0.55** to **−0.05)**	**0.02**	0.23	(−0.17–0.63)	0.26
Harm	0.04	(−0.39–0.46)	0.86	−0.10	(−0.79–0.59)	0.78
Overuse	0.28	(−0.11–0.67)	0.15	0.01	(−0.57–0.60)	0.96
Side effects	−0.84	(−1.80–0.13)	0.09	1.09	(−0.63–2.80)	0.21
Divine healing	0.06	(−1.18–1.29)	0.93	−0.79	(−2.58–1.01)	0.39
Period 2	(n = 235)			(n = 98)		
BMQ						
Necessity-concern	**0.50**	**(0.06–0.94)**	**0.03**	−0.16	(−0.74–0.41)	0.57
Necessity	**1.17**	**(0.17–2.17)**	**0.02**	−0.05	(−1.32–1.22)	0.93
Concern	−0.40	(−0.94–0.14)	0.15	0.27	(−0.49–1.03)	0.48
Harm	−0.74	(−1.58–0.09)	0.08	0.49	(−0.68–1.67)	0.40
Overuse	−0.63	(−1.37–0.12)	0.10	0.30	(−0.86–1.46)	0.61
Side effects	−2.78	(−6.61–1.05)	0.15	0.39	(−4.14–4.92)	0.86
Divine healing	**−3.73**	**(−6.50** to **−0.97)**	**0.008**	−1.50	(−5.21–2.21)	0.42

Associations statistically significant at the 5 % level shown in bold

Change in MEMS adherence associated with a 1-point higher BMQ score

### MEMS Adherence and VL Suppression (Hypothesis 2)

A significant association was observed between treatment adherence and viral suppression among children ART-naïve at enrolment (Table 5). In Period 2, each 1 % higher adherence was associated with a 5 % increase in the odds of suppression (p = 0.006), while a smaller marginally significant increase was seen in Period 1 (p = 0.07). No association was seen between adherence and viral suppression in ART experienced children, but all children were suppressed at enrolment and nearly all remained suppressed at weeks 48 and 96.

**Table 5:** Association between MEMS data and viral load suppression <100 copies/ml, adjusted for baseline characteristics

	Naive at enrolment			Experienced at enrolment		
	OR	95 % CI	p	OR	95 % CI	p
Period 1	(n = 271)			(n = 97)		
MEMs adherence (per 1 % higher)	1.03	(1.0–1.06)	0.07	0.96	(0.87–1.07)	0.49
Period 2	(n = 235)			(n = 98)		
MEMs adherence (per 1 % higher)	**1.05**	**(1.01–1.08)**	**0.006**	1.03	(0.94–1.12)	0.56

Associations statistically significant at the 5 % level shown in bold

Change in odds of viral suppression associated with a 1 % increase in MEMS

### BMQ and Viral Suppression

**Fig. 1:**
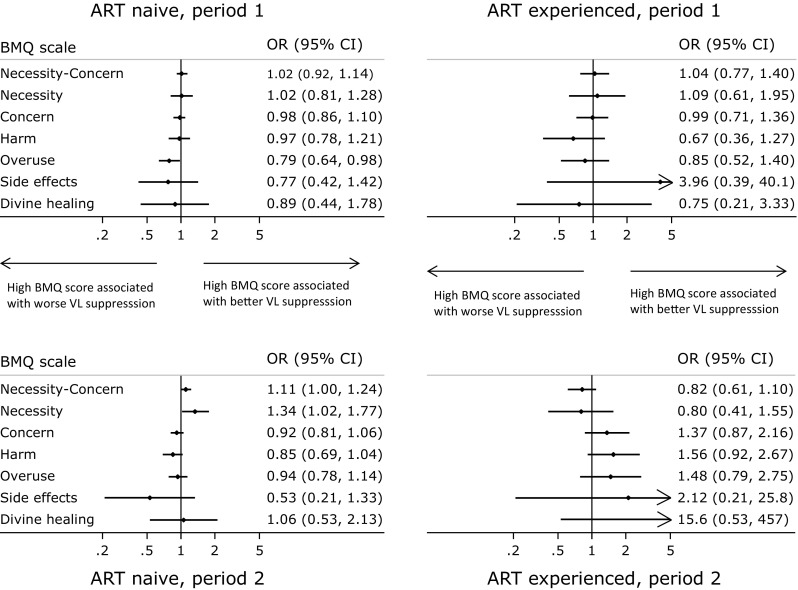
Association between BMQ scores and viral load suppression <100 copies/ml

Having found significant associations supporting hypotheses 1 and 2, we analysed the direct association between BMQ and viral suppression. A statistically significant association was observed between viral suppression and high BMQ necessity scores among ART-naïve children in Period 2 (p = 0.03), while in the same group the association between viral suppression and high necessity-concern difference scores was of marginal significance (p = 0.06; Fig. 1; Supplementary material 3). Most other associations between viral suppression and BMQ scores were not statistically significant, although there was evidence that in ART-naïve children, early suppression was less likely in children whose caregivers had greater concerns about medication (p = 0.03).

## Discussion

We observed significant associations between caregiver beliefs in medicine and their children’s adherence to treatment; children had better adherence if their caregiver’s belief in the necessity of medicine outweighed concern. We also observed significant associations between adherence measured by MEMS and suppression of viral load, confirming the results of the CHAPAS-1 study [14]. We also found some evidence of a direct association between BMQ necessity/necessity-concerns and viral load, although only in the second period, power to detect such an association being low. Further, in the initial period on ART, beliefs about overuse of medication appeared more important for viral load suppression, potentially indicating different underlying drivers of early vs later adherence in caregivers of HIV-infected children, and thus suggesting that different behaviour change techniques may need to be employed to optimise adherence and viral load response at different stages on ART.

Evidence of associations was stronger in the ART naïve group. The ART naïve group was larger than the ART experienced, so power to detect associations was higher, but the experienced children were also likely to be a more homogenous group, since the eligibility criteria requiring >2 years on ART with viral load <50 copies/ml at screening probably excluded children with poor adherence. Results were broadly similar in Periods 1 and 2. This was perhaps expected in the ART experienced group where entry to the trial represented little change. For the ART naïve, this was encouraging since long term adherence can present problems with reduced motivation to treat children regularly once their condition improves on ART [22].

The responses to the beliefs in medicine questionnaire revealed a positive overall attitude towards medicines. The necessity and concern scales, measuring attitude towards medicine in general, showed that belief in the necessity of medicines strongly outweighed concern about their use. Particularly encouraging was the increasingly positive response after 1 year on treatment, among those treatment naïve at enrolment. The harm and overuse scales, however, did show that caregivers held some concerns that they were prepared to express. Despite this, the high levels of adherence measured by MEMS caps indicated that these beliefs did not impede treatment given by caregivers, at least not to levels associated with substantially lower odds of viral suppression. To our knowledge, this is the only paediatric study conducted in sub-Saharan Africa using the BMQ to help understand children’s caregiver’s beliefs about medicines and linking them to children’s adherence and treatment outcomes.

One of the limitations of the study was that responses to the BMQ were less variable than might have been expected. The most frequent score in the necessity scale was 20, occurring when carers expressed agreement with all five questions. The lack of variability reduced ability to detect associations, and should be taken into account when interpreting the results. Another limitation is that the study population is from a clinical trial setting, where caregivers may be more highly motivated than in routine healthcare.

The negative effects on MEMS adherence observed in Period 2 among ART naïve children whose carers believed in divine healing suggest that this represents a real problem that needs to be addressed. Similarly large negative effects on adherence were associated with reporting of side-effects, although these associations did not reach even marginal statistical significance. Interestingly, no effect was seen in the ART experienced, again suggesting that this was a selected group where these issues did not present a problem. An observational study conducted in Uganda reported patients have discontinued ART due to belief in spiritual healing [23]. Therefore discussing caregiver’s beliefs/practices with reference to ART for their children may promote good adherence and avert those who may consider divine healing as quick fix to long term treatment.

Given the fact that the BMQ is a set of simple self-reported questions, the fact that any BMQ constructs were associated with the objective endpoint of viral load suppression is interesting and important. This study suggests that the BMQ necessity and necessity-concerns scores may be a good proxy for predicting adherence among children. Whilst we did not find negative associations between concerns about overuse and adherence, only negative associations between concerns and viral load suppression, it is well recognised that even MEMS is an imperfect measure of adherence, and that caregivers may open bottles but still not give the entire dose. It is certainly possible that occasionally not giving the whole dose might be more likely to occur among caregivers with concerns about overuse, and that this could influence viral load suppression. We found that the BMQ was very easy to administer to children’s caregivers. While our use was in a clinical trial setting it could be used at any level of health care, including primary health facilities and community settings. Therefore this tool could complement adherence tools already used within different health facilities, helping to identify caregivers who may benefit from more intensive follow up. Further, the fact that we identified different components of the BMQ were more strongly associated with early versus later viral load suppression in children initiating ART provides some indication as to how to target information provision more effectively at different stages on ART.

## Conclusion

We observed high levels of adherence to ART, particularly among children whose caregiver’s beliefs in necessity of treatment most strongly outweighed their concern. This study suggests that the BMQ could be a valuable tool in routine clinical practice, and that it remains important to allay concerns about overuse of medication and to ensure that caregivers are well informed about the long-term necessity of ART.

## Electronic supplementary material

Below is the link to the electronic supplementary material.

Supplementary Material 1 (DOCX 21 kb)

Supplementary Material 2 (DOCX 20 kb)

Supplementary Material 3 (DOCX 18 kb)
