# Supplementary material for: Improved Adherence to Antiretroviral Therapy Observed Among HIV-Infected Children Whose Caregivers had Positive Beliefs in Medicine in Sub-Saharan Africa
Source: AIDS Behav. 2016 Oct 19;21(2):441–9. doi: 10.1007/s10461-016-1582-8 (PMC5288435; doi:10.1007/s10461-016-1582-8)
Supplement: Supplementary file 1 — Supplementary Material 1 (DOCX 21 kb) [file 10461_2016_1582_MOESM1_ESM.docx]

**BMQ scoring.**

The BMQ contains 10 questions measuring beliefs about medicines prescribed specifically to the patient here modified to relate specifically to the caregiver’s child), and 8 questions measuring belief about medicines in general. Two further questions were added for CHAPAS-3, asking whether caregiver’s children got side effects from their treatment, and whether the caregiver believed divine healing was more important than medicine.

All questions had five possible answers: strongly disagree, disagree, don’t know, agree, strongly agree. Questions were scored from 1 to 5 (strongly disagree to strongly agree). The necessity and concern dimensions each contain five questions and scores therefore ranged from 5 to 25, while the overuse and harm dimensions contained four questions and scores ranged from 4to 20. High scores in necessity, harm and overuse indicated positive beliefs toward medicine, but a high concern score indicated a negative belief. The additional questions on side effects and divine healing were analysed separately, higher scores again indicating negative beliefs toward medicine.

The specific dimensions of the BMQ, necessity and concern, are designed to be used together to create a scale that balances a subjects belief in the necessity of their medicine against any concerns they have about them. The necessity-concern score is the difference of the two scores, taking a value between -20 and +20, with -20 indicating minimum belief in necessity and maximum concern, +20 being the opposite.
