# Supplementary material for: Improved Adherence to Antiretroviral Therapy Observed Among HIV-Infected Children Whose Caregivers had Positive Beliefs in Medicine in Sub-Saharan Africa
Source: AIDS Behav. 2016 Oct 19;21(2):441–9. doi: 10.1007/s10461-016-1582-8 (PMC5288435; doi:10.1007/s10461-016-1582-8)
Supplement: Supplementary file 2 — Supplementary Material 2 (DOCX 20 kb) [file 10461_2016_1582_MOESM2_ESM.docx]

**Use of MEMS data**

For each day that a child had a MEMS cap they were attributed 0, 1 or 2 openings. For 2 openings to count, they had to be more than 8 hours apart, and any openings beyond the second were excluded. Overall adherence during a period was then calculated using the formula:

$$Adherence= \frac{100s}{2d}$$

Where *s* is the sum of the daily doses (0, 1 or 2) and *d* is the number of days in the period. For children on once daily treatment a single opening was recoded as two openings.
