# Supplementary material for: Improved Adherence to Antiretroviral Therapy Observed Among HIV-Infected Children Whose Caregivers had Positive Beliefs in Medicine in Sub-Saharan Africa
Source: AIDS Behav. 2016 Oct 19;21(2):441–9. doi: 10.1007/s10461-016-1582-8 (PMC5288435; doi:10.1007/s10461-016-1582-8)
Supplement: Supplementary file 3 — Supplementary Material 3 (DOCX 18 kb) [file 10461_2016_1582_MOESM3_ESM.docx]

Table VI. Association between BMQ scores and viral load suppression <100 copies/ml, adjusted for baseline characteristics. Change in odds of viral suppression associated with a 1-point higher BMQ score

|  |  |  | Naïve at enrolment | | | Experienced at enrolment | | |
| --- | --- | --- | --- | --- | --- | --- | --- | --- |
|  |  |  | OR | 95% CI | p | OR | 95% CI | P |
| Period 1 | | | (n=271) | | | (n=97) | | |
|  | BMQ | |  |  |  |  |  |  |
|  |  | Necessity-Concern | 1.02 | (0.92,1.14) | 0.67 | 1.04 | (0.77,1.40) | 0.81 |
|  |  | Necessity | 1.02 | (0.81,1.28) | 0.87 | 1.09 | (0.61,1.95) | 0.76 |
|  |  | Concern | 0.98 | (0.86,1.10) | 0.68 | 0.99 | (0.71,1.36) | 0.92 |
|  |  | Harm | 0.97 | (0.78,1.21) | 0.80 | 0.67 | (0.36,1.27) | 0.22 |
|  |  | Overuse | **0.79** | **(0.64,0.98)** | **0.03** | 0.85 | (0.52,1.40) | 0.53 |
|  |  | Side effects | 0.77 | (0.42,1.42) | 0.41 | 3.96 | (0.39,40.10) | 0.24 |
|  |  | Divine healing | 0.89 | (0.44,1.78) | 0.74 | 0.75 | (0.17,3.33) | 0.71 |
| Period 2 | | | (n=235) | | | (n=98) | | |
|  | BMQ | |  |  |  |  |  |  |
|  |  | Necessity-Concern | 1.11 | (1.00,1.24) | 0.06 | 0.82 | (0.61,1.10) | 0.19 |
|  |  | Necessity | **1.34** | **(1.02,1.77)** | **0.03** | 0.80 | (0.41,1.55) | 0.50 |
|  |  | Concern | 0.92 | (0.81,1.06) | 0.24 | 1.37 | (0.87,2.16) | 0.18 |
|  |  | Harm | 0.85 | (0.69,1.04) | 0.12 | 1.56 | (0.92,2.67) | 0.10 |
|  |  | Overuse | 0.94 | (0.78,1.14) | 0.54 | 1.48 | (0.79,2.75) | 0.22 |
|  |  | Side effects | 0.53 | (0.21,1.33) | 0.18 | 2.12 | (0.17,25.80) | 0.56 |
|  |  | Divine healing | 1.06 | (0.53,2.13) | 0.88 | 15.55 | (0.53,456.5) | 0.11 |
